# The SUMO protease SENP1 promotes aggressive behaviors of high HIF2α expressing renal cell carcinoma cells

**DOI:** 10.1038/s41389-022-00440-4

**Published:** 2022-10-25

**Authors:** Moon Hee Lee, Kyung Sung, David Beebe, Wei Huang, Dan Shapiro, Shigeki Miyamoto, E. Jason Abel

**Affiliations:** 1 Department of Urology, University of Wisconsin-Madison, Madison, WI 53705 USA; 2 Division of Cellular and Gene Therapies, Office of Tissues and Advanced Therapies, Center for Biologics Evaluation and Research, the U.S. FDA, White Oak, MD 20993 USA; 3 Department of Biomedical Engineering, University of Wisconsin-Madison, Madison, WI 53705 USA; 4 University of Wisconsin Carbone Cancer Center, Madison, WI 53705 USA; 5 Department of Pathology and Laboratory Medicine, University of Wisconsin-Madison, Madison, WI 53705 USA; 6 Department of Oncology, University of Wisconsin-Madison, Madison, WI 53705 USA

**Keywords:** Renal cell carcinoma, Metastasis

## Abstract

While an important role for the SUMO protease SENP1 is recognized in multiple solid cancers, its role in renal cell carcinoma (RCC) pathogenesis, particularly the most dominant subtype, clear cell RCC (ccRCC), is poorly understood. Here we show that a combination of high HIF2α and SENP1 expression in ccRCC samples predicts poor patient survival. Using ccRCC cell models that express high HIF2α but low SENP1, we show that overexpression of SENP1 reduces sumoylation and ubiquitination of HIF2α, increases HIF2α transcriptional activity, and enhances expression of genes associated with cancer cell invasion, stemness and epithelial-mesenchymal transition. Accordingly, ccRCC cells with high HIF2α and SENP1 showed increased invasion and sphere formation in vitro, and local invasion and metastasis in vivo. Finally, SENP1 overexpression caused high HIF2α ccRCC cells to acquire resistance to a clinical mTOR inhibitor, everolimus. These results reveal a combination of high SENP1 and HIF2α expression gives particularly poor prognosis for ccRCC patients and suggest that SENP1 may be an attractive new target for treating metastatic RCC (mRCC).

## Introduction

In 2021, an estimated 76,000 adults will be diagnosed with renal cell carcinoma (RCC). The majority (~70%) of RCC is clear cell renal cell carcinoma (ccRCC), characterized by malignant epithelial cells with clear cytoplasm. Localized tumors can often be treated with surgical or ablative therapies with an estimated 5-year survival of >90% [1]. Metastatic ccRCC (mRCC) does not respond to cytotoxic chemotherapy, and systemic therapy primarily targets the VEGF or mTOR pathway or more recently includes target immune cells through the use of immune checkpoint inhibitors [2–4]. Despite advances in systemic therapy, mRCC is often lethal with a 5-year survival of only 13.9% [1]. Thus, there is a need for improving our understanding of the mechanisms regulating ccRCC metastasis and new therapeutic approaches targeting these mechanisms.

Hypoxia-inducible factor (HIF) and von Hippel-Lindau (pVHL) proteins are central to the pathogenesis of ccRCC. HIF proteins function as transcription factors composed of a heterodimer of α (HIF1α or HIF2α) and β (HIF1β) subunits. pVHL is a ubiquitin E3 ligase and enhances ubiquitination and degradation of HIF proteins [5, 6]. Clear cell RCC is characterized by the loss of pVHL expression resulting in the accumulation of active HIF proteins, leading to increased expression of their target genes. These target genes are involved in various cellular responses, such as angiogenesis, metabolism, and cell invasion. In addition to ubiquitination, other posttranslational modifications of HIFα proteins can regulate HIFα activity, such as hydroxylation, methylation, or sumoylation [7–9]. VHL, PHD1/2, and FIH in the HIF pathway regulate some of these modifications [6, 8, 10]. Understanding their mechanism of action may improve our knowledge of ccRCC pathogenesis and provide opportunities for new therapeutic targets.

Among the HIF modifications, sumoylation is of particular interest because activities of many transcription factors are regulated by SUMO (small ubiquitin-like modifier) 1, 2, and/or 3, such as the androgen receptor (AR), the tumor suppressor p53, and HIF1α [11–13]. Similar to the ubiquitination pathway, the SUMO conjugating system involves an enzymatic cascade starting with the SUMO activating enzyme (E1) followed by the SUMO conjugating enzyme (E2), and ending with the SUMO ligase (E3) which help the specificity of sumoylation by E2. SUMO modifications are reversed by SUMO-specific proteases, SENPs [11, 12, 14]. Among these, SENP1 has a broad substrate specificity and many of its substrates participate in cellular responses, such as signal transduction, cell proliferation, and apoptosis [15–17]. Consequently, SENP1 can regulate the development and metastasis of certain cancers, such as breast and prostate cancers [18, 19]. For example, sumoylation of AR suppresses its transcriptional activity, and overexpression of SENP1 in prostate cancer enhances AR transcriptional activity, promoting cancer progression and metastasis [20]. Additionally, components of sumoylation/desumoylation processes, such as SUMO E2 (ubc9), SUMO E3 (e.g., PIAS1), or SENPs (e.g., SENP1), are upregulated in various cancer types [21–23]. These data suggest that the SUMO pathway and SENP1, in particular, are critical in cancer pathogenesis.

In this study, we investigated the role of SENP1 in ccRCC. Like breast or prostate cancers that show elevated SENP1 expression, our tissue microarray (TMA) analysis found that high SENP1 expression combined with high HIF2α expression showed a poor overall survival of ccRCC patients. We established SENP1 overexpressing ccRCC cell models with high HIF2α expression and found that SENP1 overexpression reduces HIF2α sumoylation and ubiquitination and increases HIF2α transcriptional activity. It also induces the expression of genes associated with epithelial-mesenchymal transition (EMT), invasion, and cancer stemness in vitro and causes local invasion and metastatic spread in vivo. We further found that SENP1 overexpression causes increased mTOR pathway activation and resistance to mTOR inhibition. Therefore, SENP1 may be important to metastatic progression and the development of drug-resistant ccRCC. Inhibition of SENP1, specifically in HIF2α^hi^ ccRCC, might be a therapeutic approach to prevent metastasis and sensitize ccRCC to mTOR inhibitors.

## Results

### A combination of high SENP1 and HIF2α levels is prognostic for poor ccRCC patient survival

**Fig. 1:**
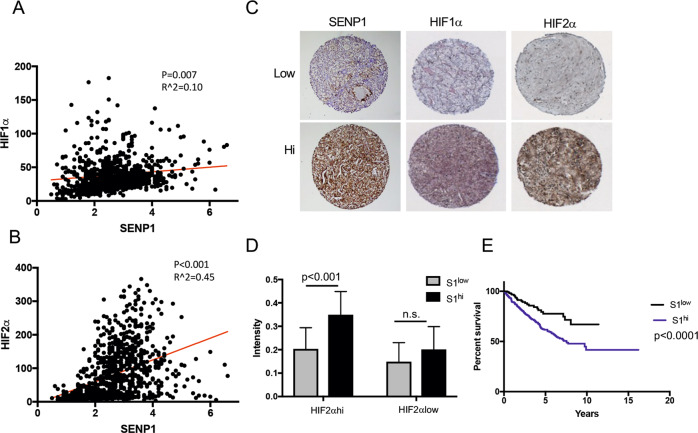
High SENP1 and HIF2α correlates with a poor ccRCC patient outcome. Spearman’s correlation of SENP1 RNA expression with HIF1α (**A**) or HIF2α (**B**) expression from the Human Protein Atlas database (FPKM, *n* = 877). Significance and correlation coefficient are shown. **C** IHC staining was performed using anti-SENP1, anti-HIF1α, and anti-HIF2α antibodies. Examples of low or high expression of each protein are shown. **D** Nuclear SENP1 staining intensities separated by nuclear HIF2α staining intensities are shown based on low or hi groups separated by the median value of all samples stained for each protein. **E** The survival rate of SENP1^hi^ or SENP1^low^ ccRCC patients with high HIF2α is shown based on staining intensities in (**D**). The error bars represent as ±SD.

It has been reported that SENP1 expression is elevated in several cancers and provides pro-tumor functions [15, 20, 24, 25]. However, the role of SENP1 in ccRCC remains unknown. Although HIF1α and HIF2α share many of the same target genes, HIF1α is postulated to act as a tumor suppressor while HIF2α has a pro-oncogenic function in ccRCC [26, 27]. Accordingly, patients with HIF1α^low^/HIF2α^hi^ ccRCCs have a worse survival rate than patients with HIF1α^hi^/HIF2α^low^ tumors [5]. To investigate the potential role of SENP1 in RCC prognosis, we analyzed SENP1 RNA expression in human RCC samples (*n* = 877) of the Human Protein Atlas database. As seen in Fig. 1A, B, SENP1 RNA expression is correlated positively with HIF2α, but not HIF1α, RNA levels in these tumor samples. To determine whether the RNA data can be extended to protein expression levels in ccRCC, we performed immunohistochemistry (IHC) analysis using anti-SENP1, HIF1α, and HIF2α antibodies with a custom tissue microarray (TMA) containing 190 benign and 471 malignant ccRCC tumor samples (Fig. 1C). Although SENP1 protein expression did not show a significant difference across disease stages 1–4 (Supplementary Fig. 1A), ccRCC with high SENP1 protein levels (S1^hi^) was significantly clustered to the high HIF2α protein expressing group (Fig. 1D). In contrast, S1^hi^ did not segregate according to HIF1α protein expression (Supplementary Fig. 1B). When we analyzed patient survival, we found that a combination of HIF2α^hi^ and S1^hi^ had significantly poorer patient survival relative to HIF2α^hi^ but S1^low^ patients (Fig. 1E). However, a similar analysis showed a combination of HIF1α and SENP1 levels did not significantly correlate with patient survival (Supplementary Fig. 1C). Thus, our results suggest that a combination of HIF2α^hi^ and SENP1^hi^ status is a new prognostic marker for poor ccRCC patient survival.

### SENP1 overexpression does not increase ccRCC cell growth in vitro and in vivo

**Fig. 2:**
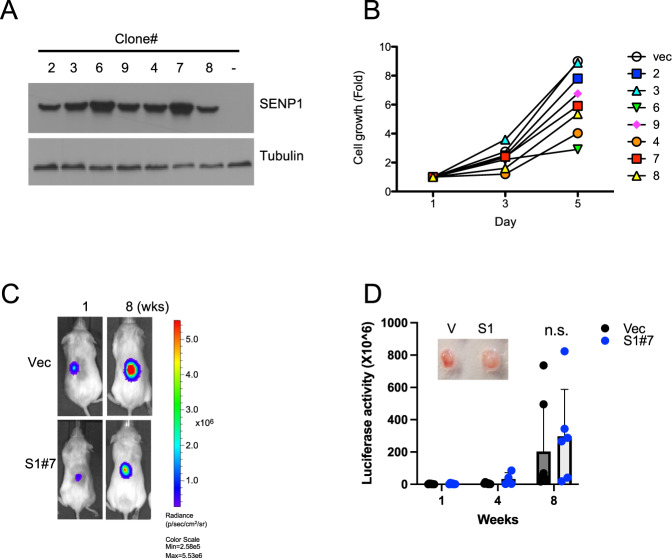
SENP1 overexpression does not increase the proliferation of 786-O ccRCC cells. **A** Immunoblots showing 786-O clones stably expressing SENP1 or vector control (-). **B** A graph showing fold change in the growth of SENP1 overexpressing 786-O cell clones over 5 days relative to vector control cells. **C** 786-O/Luciferase cells (vector control or S1#7 clone) were injected into the flank of 6–8 weeks old NSG mice. Mice were subjected to imaging by IVIS spectrum (PerkinElmer) using luciferin as the substrate. **D** Bar graph showing the luciferase activity from mice bearing tumors established from indicated 786-O cell clones, *n* = 7 for each cohort. Inset shows the images of representative tumors.

To investigate the functional consequence of high SENP1 expression in HIF2α^hi^ ccRCC, we next examined SENP1 and HIF2α expression in various ccRCC cell lines in order to identify an appropriate cell model. Expression of HIF2α was high in 786-O cells relative to other ccRCC cells but with low SENP1 expression (Supplementary Fig. 1D, E). 786-O cells have been reported to express only HIF2α protein without expression of functional HIF1α or pVHL [28]. Thus, we chose 786-O cells as a model to study the impact of high SENP1 expression in the context of high HIF2α and low or no HIF1α expression. Next, we generated multiple SENP1 overexpressing 786-O cell clones (Fig. 2A) and tested their proliferation potentials over five days in culture (see Methods). Interestingly, SENP1 overexpressing ccRCC cell clones grew at a slower rate relative to the vector control clone (Fig. 2B). To enable monitoring of in vivo growth of SENP1 overexpressing ccRCC cells, we further generated luciferase-expressing stable cells using control cells or SENP1-overexpressing 786-O cell clone because of its intermediate proliferation phenotype (Fig. 2B). 1 × 10^6^ such cells were suspended in 50% matrigel and injected subcutaneously in the flank of immunocompromised NSG (NOD.*Cg-Prkdc*^*scid*^ *Il2rg*^*tm1Wjl*^/SzJ) mice. The tumor growth was monitored by bioluminescence imaging every 2 weeks for a total of 8 weeks. The growth in SENP1 overexpressing tumors was not significantly different from the vector control cells (Fig. 2C, D). Western blot analysis showed that SUMO1 modification levels were lower in ccRCC tumors derived from SENP1 overexpressing cells relative to control cells (Supplementary Fig. 2A), thus confirming SENP1 was active in the tumors. Thus, SENP1 overexpression did not cause increased proliferation of these ccRCC cells in vitro or in vivo.

### SENP1 induces desumoylation of HIF2α in ccRCC cells

**Fig. 3:**
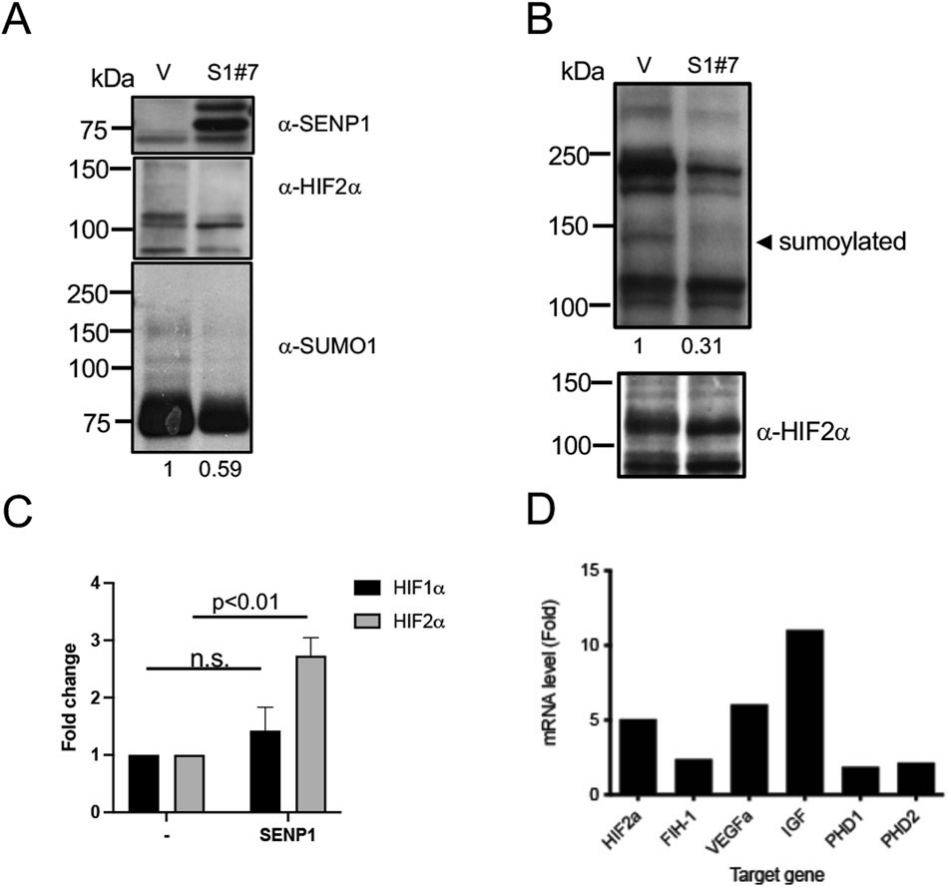
SENP1 overexpression alters HIF2α sumoylation. **A** SNEP1-overexpressing 786-O cells (S1#7) and vector control cells (V) were lysed in IP buffer and analyzed by immunoblotting with the indicated antibodies. **B** The samples as in **A** were denatured by boiling in 1% SDS buffer and processed for IP analysis with anti-HIF2α antibody and sumoylation of HIF2α was assessed by immunoblotting using anti-SUMO1 antibody. The numbers under immunotblots represent the quantification of total sumoylated proteins above the major ~75 kDa band (A) or a sumoylated HIF2α (arrowhead pointed). **C** A HRE-Luc reporter was transfected into parental 786-O cells with HIF1α, HIF2α, or SENP1 expression constructs, and the resulting luciferase activity was measured after 48 h. The graph shows the fold change of luciferase activity normalized by the activity of HIF1α or HIF2α alone transfected cells. This was performed as duplicates, three-independent expreiments, and represented as ±SEM. **D** qRT-PCR analysis of indicated genes was performed using SENP1-overexpressing (S1#7) cells. The graph shows the fold change of the expression of indicated genes in SENP1-overexpressing cells relative to vector control cells.

Next, we investigated the status of HIF2α in SENP1 overexpressing 786-O ccRCC cell clones. Western blot analysis revealed that HIF2α protein appeared to migrate as multiple higher molecular weight bands in the control cells; however, in SENP1 overexpressing cells (S1#7), a lowest molecular weight band was dominant (Fig. 3A). The latter was also accompanied with reduced overall sumoylation (Fig. 3A, lower panel). To test whether the HIF2α protein pattern seen above was due to sumoylation, HIF2α was immunoprecipitated after denaturation of cell extracts and probed with SUMO1 antibody. The result demonstrated that sumoylated HIF2α bands were detected in the control cells but was overall reduced in SENP1 overexpressing cells (Fig. 3B), thus confirming that SENP1 overexpression reduces SUMO1-modified HIF2α levels in ccRCC cells. Next, we assessed whether the expression of SENP1 affected HIF2α transcriptional activity by means of hypoxia-response element (HRE)-luciferase reporter assay. Indeed, SENP1 overexpression significantly increased HIF2α transcriptional activity (Fig. 3C). In contrast, SENP1 did not increase HIF1α activity. This correlated with higher SENP1 and HIF2α interaction relative to HIF1α interaction (Supplementary Fig. 2B). Accordingly, the expression of known HIF2α target genes was also increased 2–12-fold in the SENP1 overexpressing ccRCC cells (Fig. 3D). Thus, SENP1 overexpression increased HIF2α activity in association with its reduced sumoylation in ccRCC cells.

### SENP1 overexpression induces genes related to cell morphogenesis, invasion, and stemness in ccRCC cells

**Fig. 4:**
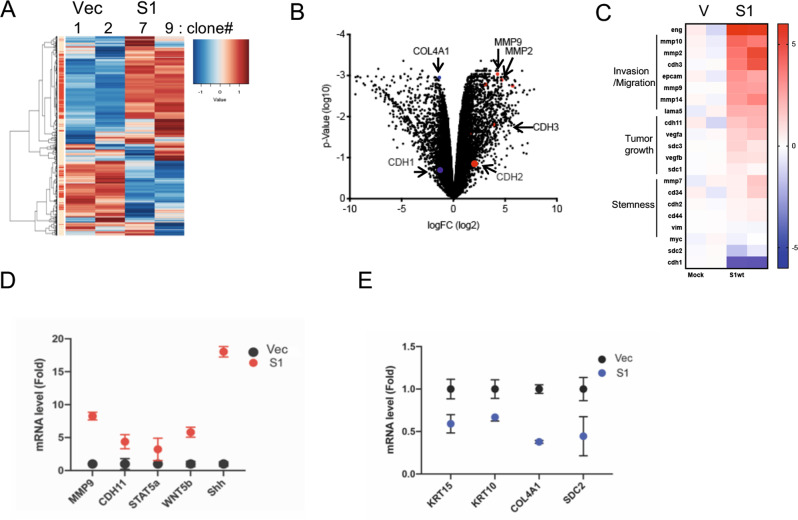
SENP1 overexpression increases the expression of cell invasion-related genes in 786-O cells. he heatmap (**A**) and volcano graph (**B**) of RNA-seq data from two different ccRCC cell clones for each group (vector or SENP1 overexpressing cells) are shown for mRNA expression with ≥2-fold changes with significance at (*p* < 0.05). The red and blue dots in the volcano graph highlight several genes displaying differences. **C** Heat map showing clusters of malignancy-related genes from RNA-seq analysis from (**A**). mRNA levels of select genes from **C** were analyzed by qRT-PCR. The graphs show the fold change as mean ±SEM. RNAs increased are shown in red (**D**) and those decreased are shown in blue (**E**).

To unbiasedly gain insight into potential functional alterations induced by SENP1 overexpression in ccRCC cells, we next performed RNA-seq analysis of SENP1 overexpressing 786-O cells clones and vector-control clones. Each sample was well-clustered based on GSEA analysis (Fig. 4A). In SENP1 overexpressing cells, the expression of 1452 genes were significantly increased by two-fold or greater, while 773 genes were reduced relative to the vector transfected control cells (*p* < 0.05, Fig. 4A and B). Among upregulated genes, the major group was related to cellular morphogenesis (Supplementary Fig. 3A), invasion/migration and stemness (Fig. 4C), as well as stem cell differentiation and WNT signaling that also relates to stemness (Supplementary Fig. 3B). We confirmed the induction of select genes detected by RNA-seq analysis in SENP1 expressing cells (S1#7) by qRT-PCR analysis (Fig. 4D, E).

### SENP1 induces invasion, epithelial-mesenchymal transition, and metastasis of ccRCC cells

**Fig. 5:**
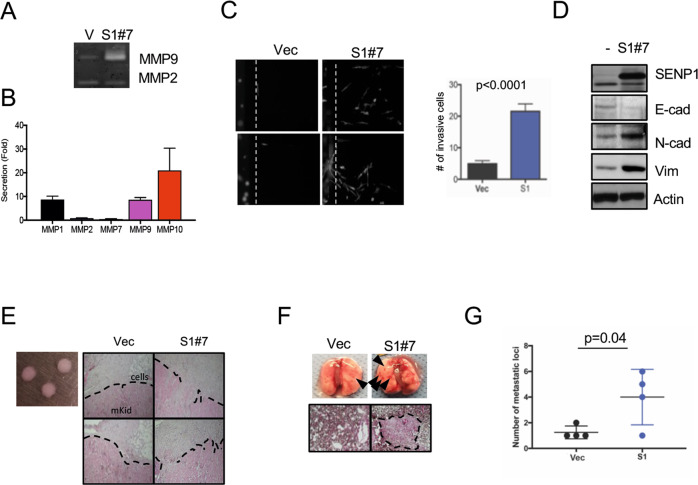
SENP1 overexpression increases EMT-related proteins, invasion, and metastasis of ccRCC cells. **A** 10^6^ Control or SENP1-overexpressing (S1#7) 786-O cell clones were plated in serum-free media. After 24 h, the conditioned media from these cells were collected and subjected to 0.1% gelatin gel zymography assay. MMP9 and MMP2 activities were visualized by staining with the Coomassie blue. **B** The above-conditioned media from SENP1-overexpressing cells was collected and used for ELISA-based Magplex assay (Luminex). The graph shows secreted amounts of several MMPs in SENP1 overexpressing cells relative to control cells. **C** Indicated cells were mixed with collagen and matrigel and loaded into the microchannels. After 3 days, cells were fixed and stained with phalloidin-rhodamine. Eight independent SENP1-expressing 786-O cell clones were analyzed, and the assay was repeated in three biological replicates with each performed in duplicate. The number of migrating cells was plotted in the bar chart. **D** The ccRCC cell samples (S1#7) in **C** underwent immunoblot assay for the indicated proteins. **E** 3.5 × 10^5^ of indicated tumor cells (vector or S1#7) in collagen (left panel) were grafted under the kidney capsule of NSG mice. One month later the kidneys were fixed and processed for H&E staining. The dotted line denotes the edge of injected tumor cells (pale pink) from the mouse kidney (pink). **F** Tumor cells (vector or S1#7) were injected into the tail vein of NSG mice. After 2 months, the lungs from corresponding mice were harvested and analyzed for the presence of metastatic tumors. The metastatic tumors in the lung are shown in the gross specimens by the arrows (upper panels) and the dotted line in H&E staining (lower panels). **G** Number of metastatic foci per lung (*n* = 4 for each group) from **F** were plotted.

The above RNA-seq analysis found increased expression of many MMPs that can improve the invasive potential of tumor cells, and vimentin and N-cadherin that are linked to epithelial-mesenchymal transition (EMT) [18, 29] in 786-O cells overexpressing SENP1. Invasiveness and EMT are critical for cancer metastasis [18]. Thus, we investigated whether SENP1 overexpression causes increased secretion of MMPs, invasion, and metastasis of ccRCC cells. SENP1 overexpression increased active MMP9 in conditioned media as detected by gelatin zymography (Fig. 5A and Supplementary Fig. 4A) and secretion of several other MMPs as measured by ELISA (Fig. 5B). Next, invasion of ccRCC cells was examined by a matrigel- and microchannel-based invasion assay [30] in which cancer cells were placed in matrigel and tracked by actin-phalloidin staining. The invasion of these cancer cells was also enhanced by SENP1 expression (Fig. 5C and Supplementary Fig. 4B). We found increased expression of N-cadherin (CDH2), a marker of mesenchymal cells, and decreased expression of E-cadherin (CDH1), a marker of epithelial cells, in the RNA-seq analysis (Fig. 4B). Western blot analysis confirmed that the expression of N-cadherin and vimentin was increased in SENP1 overexpressing cells, while E-cadherin was reduced (Fig. 5D). To test ccRCC tumor invasion in vivo, we mixed 3.5 × 10^5^ SENP1-overexpressing or control cells with neutralized collagen and implanted them orthotopically under the kidney capsules of NSG mice. Histological analysis of the tumor boundaries showed that implanted SENP1-overexpressing ccRCC cells showed invasion of tumor cells under the mouse kidney capsules, which was undetectable in tumors derived from the control cells (Fig. 5E). Finally, the metastatic potential of SENP1-overexpressing ccRCC cells was measured by injecting them into NSG mouse tail veins and assessing lung metastasis after 2 months. Gross examination of lungs demonstrated the development of more metastatic lung tumors with SENP1-overexpressing cells (Fig. 5F) compared to the control cells. Serial histological sections confirmed the presence of a significantly higher number of metastatic tumor foci in the SENP1 overexpressing group (Fig. 5G). These results demonstrate that SENP1 overexpression in HIF2α^hi^ ccRCC cells is also associated with higher invasive and metastatic potential in vivo.

### SENP1 overexpression increases stemness and confers resistance to an mTOR inhibitor in ccRCC cells

**Fig. 6:**
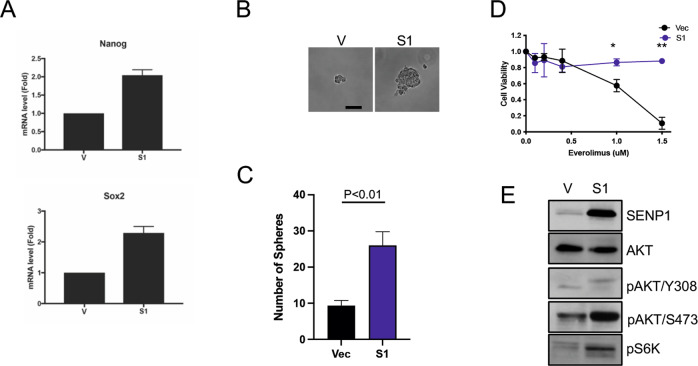
SENP1 overexpression increases the stemness of ccRCC cells and confers resistance to mTOR inhibition. **A** Messenger RNA levels for Nanog (upper) and Sox2 (lower) were measured by qRT-PCR in control (V) and SENP1 overexpressing 786-O cells (S1#7) and plotted. 200 cells of control (V) or SENP1-expressing clone (S1#7) were seeded on low attachment plates in sphere-forming assay media. After 14 days, representative pictures of the spheres were taken (**B**) and the number of spheres were counted in three replicas of three independent experiments (**C**). **D** Luciferase-expressing 786-O cells (vector and S1#7) were exposed to increasing doses of everolimus for 24 h, and the luciferase activity in the remaining viable cells was measured by a luminometer. **E** 786-O cells (vector and S1#7) cells were analyzed by immunoblotting using the indicated antibodies.

Recent studies demonstrate that SENP1 enhances the stemness of certain tumor cells, such as certain hepatocellular carcinoma and prostate cancer cell lines [25, 31]. Our RNA-seq analysis also found higher expression of certain genes that are linked to stemness, such as WNTs, CD44, Nanog, and Sox2 (Fig. 4C and Supplementary Fig. 3B). Indeed, we found that SENP1-overexpressing ccRCC cells had higher expression of CD44 (Supplementary Fig. 5), and Nanog and Sox2 (Fig. 6A) relative to the control cells. To investigate whether overexpression of SENP1 increases the stemness of ccRCC cells, we performed a sphere-forming assay that detects the clonogenicity of cancer stem cells [25]. Cells were plated in low attachment dishes and cultured in sphere-forming media for 10 days. SENP1 overexpression resulted in a significantly increased number and size of spheres formed by ccRCC cells (Fig. 6B, C). Thus, these results indicated that SENP1 overexpression increased cancer stemness in ccRCC cells.

Clear cell RCC tumors are notoriously resistant to conventional chemotherapies [2, 3] and cancer stem cells are often associated with increased drug resistance [32]. Our TMA and bioinformatic analyses also found that ccRCC patients with higher expression of SENP1 and HIF2α had worse overall survival (Fig. 1E). Given that SENP1 overexpression caused increased cancer stemness, we also tested whether SENP1 overexpression caused resistance to drugs that are currently used to treat ccRCC. SENP1 overexpressing cells showed near complete resistance to everolimus, an inhibitor of mTOR pathway, up to 1.5 µM where >90% of the control cells showed loss of viability (Fig. 6D). Moreover, SENP1 overexpression was associated with increased mTOR pathway activity as measured by immunoblot analysis of key pathway components, such as pAKT and pS6K (Fig. 6E). It has been reported that mTOR pathway is critical in stemness of cancer cells as well [33]. Thus, SENP1 overexpression caused mTOR pathway activation, increased cancer stemness, and resistance to the mTOR inhibitor.

Overall, SENP1 overexpression caused more malignant phenotypes in HIF2α^hi^ ccRCC cells, including increased HIF2α transcriptional activity, invasion, EMT, stemness, metastasis, and resistance to mTOR inhibitor. These results identify SENP1 as an important pathogenic factor in HIF2α^hi^/SENP1^hi^ ccRCC and a potential biomarker and therapeutic target in ccRCC patients with particularly poor prognosis.

## Discussion

In the present study, we found that combined high expression of SENP1 and HIF2α (SENP1^hi^/HIF2α^hi^) is a poor prognostic marker for patients with clear cell renal cell cancer (ccRCC). Moreover, SENP1 overexpression in HIF2α^hi^ ccRCC (786-O) cells increased their in vitro and in vivo invasion, metastasis, stemness, and resistance to mTOR inhibitors. ~70% of RCC patients exhibit deletions or loss-of-function mutations of the *vhl* gene [5, 34, 35], which is a tumor suppressor and functions as a ubiquitin E3 ligase of HIF1α and HIF2α proteins. Thus, a functional deficiency of VHL results in the accumulation of HIF proteins and induction of various cellular responses [6, 27, 36]. While a loss of VHL function is one of the major events for RCC pathogenesis, it does not capture the heterogeneity of RCC subtypes and how they become resistant to specific systemic therapies. Our results demonstrate that SENP1 overexpression, specifically in the setting of high HIF2α expression, may be a new pathogenic mechanism for ccRCC progression.

The HIF2α protein is known to be modified by SUMO1 [9], and we accordingly found that SENP1 overexpression induced desumoylation of HIF2α. We additionally showed that SENP1 overexpression increased HIF2α transcriptional activity. Therefore, SENP1 enhances the activity of the HIF2α pathway, a known contributor to poor prognosis in ccRCC. We also found that SENP1 increases transcriptional activity of HIF2α but not HIF1α in 786-O ccRCC cells, which was correlated with SENP1 interacting with HIF2α better than HIF1α. In recent studies, HIF2α has been postulated to act as an oncoprotein, while HIF1α functions as a tumor suppressor in RCC [26]. Thus, HIF1α^low^/HIF2α^hi^ RCC patients show worse survival compared to those with HIF1α^hi^/HIF2α^hi^ tumors. We found that the SENP1^hi^ status poses a worse prognosis than SENP1^low^ within HIF2α^hi^ ccRCC. Thus, the high SENP1^hi^ status in HIF2α^hi^ ccRCC cases may be causally related to reduced sumoylation of HIF2α induced by SENP1 thereby resulting in higher HIF2α pro-malignant activity.

SENP1 overexpression has been previously reported to enhance tumor growth by desumoylating components of signaling pathways relevant to breast or prostate cancers [31, 37, 38]. Dong et al. also found that SENP1 overexpression increased the proliferation of HIF1α^hi^/HIF2α^hi^ ccRCC4 cells [39, 40]. The authors suggested that this proliferation effect of SENP1 was mediated via HIF1α, and the expression of HIF1α was positively correlated with SENP1, but not HIF2α. In contrast, we found that SENP1 overexpression in HIF2α^hi^ and HIF1α-deficient 786-O cells does not cause increased proliferation in vitro or in vivo. Instead, this promoted the growth of cancer stem cells without affecting the proliferation of bulk cancer epithelial cells. We also performed the sphere forming assay with SENP1-overexpressing ACHN cells, a HIF1α^hi^ and HIF2α^hi^ ccRCC cell line, and found no increase in MMP9 and sphere forming activities (Supplementary Fig. 6A–C). Our data suggest that SENP1 expression in HIF2α^hi^/HIF1α^lo^ cells might enhance the stemness of tumor cells without increasing the proliferation of overall tumor epithelial cells.

SENP1 overexpression not only increased stemness but also caused increased invasion and metastasis of 786-O ccRCC cells. In RNA-seq data, SENP1 induced many genes related to epithelial-mesenchymal transition (EMT) and invasion processes in these HIF2α^hi^ ccRCC cells, such as vimentin and MMPs, respectively. Indeed, SENP1 overexpression enhanced invasion of 786-O cells in vitro. SENP1 also increased the invasion of 786-O cell xenografts in kidneys and metastasis to lung in vivo. These results suggest that SENP1 enhances the invasion and metastatic potential of HIF2α^hi^ ccRCC. Thus, our results highlight the possibility of SENP1 inhibition as a potential therapeutic approach to inhibit metastasis of HIF2α^hi^/SENP1^hi^ ccRCC cases.

SENP1 expression might also increase the resistance of HIF2α^hi^ ccRCC cells to mTOR pathway inhibitors. SENP1-overexpressing cells showed less sensitivity on treatment with everolimus, an inhibitor of mTOR pathway. One mechanism of resistance could be due to increased expression of mTOR pathway proteins and activation by SENP1 overexpression. Prior studies have reported SENP1-mediated desumoylation of various components, like the regulatory subunit GβL, AMPK, or LKB, which regulates the mTOR pathway directly or indirectly [5, 41, 42]. Similarly, we found that the phosphorylation of AKT and S6K was increased by SENP1 overexpression in 786-O cells. Although inhibitors of the mTOR pathway, such as everolimus, are used for the treatment of RCC, mTOR inhibitors do not achieve impressive clinical outcomes compared to modern immune checkpoint inhibitor therapies. Our study suggests the possibility that combined inhibition of SENP1 and mTOR could induce a more favorable outcome for SENP1^hi^/HIF2α^hi^ ccRCC cases. Further studies are however required to determine whether SENP1 overexpression in HIF2α^hi^ ccRCC cells also modifies cancer sensitivities to other therapeutic agents used in the clinic. Despite this need, our study identifies SENP1 as a new biomarker as well as a new therapeutic target for HIF2α^hi^ ccRCC patients with particularly poor clinical outcomes.

## Methods

### Cells, antibodies, and reagents

HEK293 cells were grown in DMEM medium and ccRCC cell lines (786-O, ACHN, M48, and M62) cells were grown in RPMI/MEM medium supplemented with 100 units/ml of penicillin, 1 μg/ml of streptomycin, and 10% FBS in 5% CO_2_ incubator at 37 °C. 786-O and ACHN cells were purchased from ATCC and used in 5 passages. To generate SENP1 expressing 786-O cell clones, pCMV-3Flag-SENP1 construct was transfected, and cells were selected by treatment of puromycin (1 μg/mL) for several weeks. Specific clones were isolated by isolating individual colonies. The control cells were also generated in parallel by transfecting 786-O cells with the empty vector, selecting with puromycin and isolating resistant clones as above. For bioluminescence imaging, pGL4-Luc2 (Promega) was transfected into control 786-O cell clone or S1#7 clone and luciferase-expressing stable cell pools were selected with hygromycin B (200μg/mL).

Antibodies used for TMA and immunoblot assays were from Abcam - anti-HIF1α (ab51608), anti-HIF2α (ab109616), anti-SENP1 (ab108981); Sigma Aldrich - anti-Flag (M2): Roche - anti-HA (3F10): and Cell Signaling - anti-Vimentin (D21H3), anti-E-cadherin (4A2), anti-N-cadherin (D4R1H), phospho-AKT (193H12), and phospho-S6K (49D7). Anti-SUMO1 (GMP1) antibody was purchased from Invitrogen and purified from the supernatant of hybridoma cells. For flow cytometry, anti-CD44-FITC (ab19622) was purchased form Abcam. Everolimus was purchased from Selleckchem, LLC.

### Public RNA data and tissue microarray (TMA) analyses

To investigate the correlation of expression of HIF’s and SENP1 genes, the publicly available Human Protein Atlas database of RCC samples (*N* = 877) were analyzed for the correlation (R^2) or covariation by Spearman correlation methods for RNA expression of HIF1α, HIF2α, and SENP1 genes. For tissue microarray (TMA) analysis, malignant and tumor-adjacent benign tissues were used to construct a manual tissue array [43]. A total of 471 malignant cores and 190 benign cores were included. Immunohistochemistry was performed by UW Translational Research Initiatives in Pathology (TRIP) facility. TMA with human samples was performed the protocol (#2011-0179) approved by IRB. Anti-SENP1 (ab108981), anti-HIF1α (ab51608), or anti-HIF2α (ab109616), all from Abcam, MA, was applied to the TMAs, and hematoxylin was used for count-staining. Stained slides were scanned by Vectra slide scanner (PerkinElmer, MA) and SENP1, HIF1α, and HIF2α expression levels in the nuclears (where SENP1 would act on these HIF proteins) were quantified and analyzed in inForm software (PerkinElmer).

### In vitro proliferation assay

2 × 10^4^ cells were plated in each well of six-well plates. Cells were grown in the above RPMI culture media, fixed and stained by 2.5% crystal violet solution after 1, 3, or 5 days in culture. The dye was resolved with 50% methanol and measured by a spectrophotometer (540 nm).

### In vivo tumor growth and Bioluminescence imaging (BLI)

10^6^ 786-O cells (S1#7 or control cell clone) stably expressing luciferase were mixed with 50% Matrigel (BD sciences) and injected into the side flank (subcutaneous injection) of 6–8 weeks old NOD *scid* gamma (NSG) mice (NOD.*Cg-Prkdc*^*scid*^ *Il2rg*^*tm1Wjl*^/SzJ). To measure the growth of injected cells, 2 mg of D-luciferin in 100 μl of 20 mg/ml solution was injected intraperitoneally into each mouse and visualized after 5 min by bioluminescence image (BLI) instrument (IVIS, PerkinElmer). BLI images were taken every 2 weeks for 2 months. There is no randomization and blinding experiments. All animal experiments were performed under a protocol (#M005757) approved by IACUC.

### Immunoprecipitation

To examine the modifications of HIF2α, cells were lysed by SDS-IP buffer composed of 1% SDS, 3% glycerol and Tris-HCl (pH 6.8), along with 1X protease inhibitor cocktail (Invitrogen). The samples were boiled for 10 min and diluted 10-fold by adding immunoprecipitation (IP) buffer (50 mM Tris-HCl, pH 7.4, 120 mM NaCl, and 0.5 % NP-40). The supernatant was used for IP with anti-HIF2α antibody and sumoylation of HIF2α was analyzed by immunoblotting with anti-SUMO1 antibody (GMP1). For co-immunoprecipitation of SENP1 and HIF1α or HIF2α, HEK293 cells were transfected with HA-tagged HIF1α, HA-tagged HIF2α, or Flag-tagged SENP1 vectors, and cell lysates were made at 24 h after transfection. Anti-Flag antibody (M2) was used to immunoprecipitate Flag-SENP1 complexes and anti-HA and flag antibodies were used for immunoblot analysis. Band intensities were quantified by ImageJ software.

### RNA-seq and qRT-PCR analyses

Total RNAs were prepared by Nucleospin RNA kit (Macherey-Nagel Inc, PA). After reverse transcription, the resulting cDNA was used for quantitative RT-PCR experiments using the primers described in Supplementary Fig. 7. RNA-seq integrated workflow service was provided by ProteinCT Biotechnologies (Madison, WI). For library preparation, total RNA was isolated from 786-O vector control (clone #1 and #2) and SENP1-overexpressing cells (clone #7 and #9) using TRIZol reagent (Life Technologies), and mRNA libraries were prepared using the Illumina TruSeq strand-specific mRNA sample preparation system (Illumina). The libraries were sequenced (Single end 100 bp reads) using the Illumina HiSeq4000, a final of around 30–40 million reads per sample. The fastQC program was used to verify the raw data quality of the Illumina reads. The hg19 human genome and Ensembl gene annotations (v75) were used for mapping. The raw sequence reads were mapped to the genome using Subjunc aligner from Subread, with the majority of the reads (over 96% for all samples) aligned to the genome. The alignment bam files were compared against the gene annotation GFF file, and raw counts for each gene were generated using the feature. Counts tool from Subread, with 84–87% of reads overall assigned to genes. The raw counts data were normalized using voom method from the R Limma package, then used for differential expression analysis. The normalized data were analyzed for their clustering groups by GSEA and MSigDB software (UCSD and Broad Institute) [44].

### Gelatin zymography

10^5^ cells were plated in each well of 6-well plates and the growth media were replaced with the same media without serum the next day. After 24 h, the conditioned media were collected and subjected to 0.1% gelatin/SDS-PAGE under non-reducing conditions and without sample boiling prior to gel elecrtophoresis. The gel was renatured by incubating in Tris-HCl buffer (50 mM Tris-HCl pH7.4) and incubated in reaction buffer (50 mM Tris-HCl pH7.4, 200 mM NaCl, and 10 mM CaCl_2_) for 16 h, at 37 °C. After incubation, the gel was stained with Coomassie R-250 solution and incubated with destaining buffer (10% Acetate, 40% Methanol, and 50% water). The clear bands were used for the measurement of activity of MMP2 or MMP9 by ImageQuant program.

### Quantification of secreted MMPs

To measure the amount of secreted MMPs, 10^6^ 786-O cells were incubated with serum-free RPMI1640 media for 24 h. The collected conditioned media were incubated with the mixture of microbeads conjugated specific antibodies, such as MMP1/2/7/9/10. After incubation for 1 h, the samples followed washing steps as per the manufacturer’s protocols. The beads previously dyed with distinct spectral sets were measured individually by an xMAP instrument (Luminex, IL, USA).

### In vitro invasion assay

2000 cells were mixed with collagen (1 mg/ml) and 25% Matrigel in serum-free RPMI1640 media. Cells were loaded to microchannels for invasion assay as described in refs. [45, 46]. This microchannel is designed for invasion assay and has 2 channels linked to a microchannel between them. One side was loaded with cells in gel and the other side was loaded with complete growth media for inducing cell movement by chemotaxis. After 3 days, cells were fixed with 4% PFA and stained with phalloidin-rhodamine (Abcam, MA), and migrated cells out of the gels were counted manually using a microscope.

### In vivo tumor invasion assay

For in vivo invasion assay, 3.5 × 10^5^ above cells were mixed with neutralized collagen and placed under kidney capsule (orthotopic injection) of 6–8 weeks old NSG mice. After 2 months, kidneys were isolated and analyzed by H&E staining for invasion of ccRCC tumors.

### Sphere forming assay

For the growth of cancer stem cells, 200 cells (luc-expressing 786-O) were plated in each well of 24-well low-attachment plates (Nunc) with sphere-forming media (MEM media supplemented with 1% penicillin-streptomycin, 20 ng/ml human EGF, 20 ng/ml human basic FGF, and 1X B27) [47]. Cells were supplied with fresh media every three days for 10–14 days. Visible spheres were counted under a microscope and the lysate from these spheres was measured for the luciferase activity by a luminometer.

### Drug toxicity assay

2 × 10^4^ luciferase-expressing 786-O cells were seeded in 96-well plate with various doses of drugs (0–1.5 μM). After 24 h, cells were harvested, and luciferase activity was measured using the substrate luciferin.

## Supplementary information

Supplementary figure legends

Supplementary Fig 1

Supplementary Fig 2

Supplementary Fig 3

Supplementary Fig 4

Supplementary Fig 5

Supplementary Fig 6

Supplementary Fig 7

## Data Availability

All supporting data are available in the article and its supplementary information. For the data of RNA-seq, you can access and edit through the public open source. To view the data, visit 10.6084/m9.figshare.20001461.
