# Supplementary material for: The SUMO protease SENP1 promotes aggressive behaviors of high HIF2α expressing renal cell carcinoma cells
Source: Oncogenesis. 2022 Oct 25;11(1):65. doi: 10.1038/s41389-022-00440-4 (PMC9596416; doi:10.1038/s41389-022-00440-4)
Supplement: Supplementary file 1 — Supplementary figure legends [file 41389_2022_440_MOESM1_ESM.doc]

**SUPPLEMENTARY FIGURE LEGENDS**

**Supplementary Fig. 1. SENP1 expression in ccRCC samples from different disease stages and ccRCC cell lines.**

**A** Median nuclear SENP1 staining intensities in ccRCC samples from different disease stages based on IHC analysis of TMA shown in Fig. 1C was plotted. **B** Nuclear SENP1 staining intensities separated by nuclear HIF1α staining intensities are shown based on low or hi groups separated by the median value of all samples stained for each protein. **C** The survival rates of SENP1^hi^ or SENP1^low^ ccRCC patients within the HIF1α^hi^ cohort are shown. **D** Messenger RNA levels of SENP1, 2, and 3 in HEK293, 786-O, and ACHN cell lines were measured by qRT-PCR and plotted. **E** Immunoblots of HIF2α, SENP1 and Tubulin (loading control) in HEK293 (293), 786-O, ACHN, MDA-RCC-48 (M48), and MDA-RCC-62 (M62) cell lines are shown.

**Supplementary Fig. 2. Reduced SUMO-1 modification in SENP1-overexpressing ccRCC tumor xenografts and SENP1 interaction with HIF2α versus HIF1α.**

**A** NSG mice were injected with vector or SENP1 overexpressing 786-O RCC cell clone (#7) and after 8 weeks tumor extracts were analyzed for SUMO1 conjugated proteins by anti-SUMO1 immunoblot analysis. The numbers below show relative amounts of modified proteins. **B** HEK293 cells were transfected with HA-HIF1α or HA-HIF2α and Flag-SENP1 as indicated. Cell extracts were used to perform IP assays with anti-Flag antibody and analyzed by immunoblotting for indicated proteins. The band intensities were quantified by ImageJ software and HIF1α and HIF2α binding was normalized to SENP1 IP levels.

**Supplementary Fig. 3. GSEA analysis of RNA-seq data**

**A-B** Differentially expressed genes in the RNA-seq dataset of SENP1-overexpressing cells relative to control cells were clustered based on biological processes (**A**) or phenotype database (**B**) by gene set enrichment analysis (GSEA).

**Supplementary Fig. 4. Increased MMP9 activity and invasion in SENP1-expressing ccRCC cell clones.**

**A** Conditioned media from parental (-), vector-transfected (V) or SENP1 overexpressing 786-O ccRCC cell clones were analyzed by gelatin zymography. The locations of MMP9 and MMP2 are indicated. **B** Indicated SENP1 expressing 786-O cell clones were assessed by a microchannel-based invasion assay as in Fig. 5C. The stained invaded cells were counted. The bar indicates the median value of replicas shown in dots for each clone.

**Supplementary Fig. 5. CD44 expression is increased in SENP1 overexpressing ccRCC cells.**

Vector (V) or SENP1-overexpressing 786-O cell (#7) clones were stained with anti-CD44 antibody or control IgG (vector cells) and analyzed by flow cytometry.

**Supplementary Fig. 6. Effects of SENP1 expression in ACHN cells.**

**A** MMP9 activity from serum-free conditioned media of vector control or SENP1-overexpressing ACHN ccRCC cell clone (#1) was analyzed by gelatin zymography. **B** The sphere forming assay with above ACHN cells was performed for 14 days. Examples of spheres are shown. **C** The number of spheres from **B** was measured and plotted. n.s.: not significant.

**Supplementary Fig. 7**

A list of primers used for qRT-PCR analysis.
