# Supplementary figures and images for: The SUMO protease SENP1 promotes aggressive behaviors of high HIF2α expressing renal cell carcinoma cells
Source: Oncogenesis. 2022 Oct 25;11(1):65. doi: 10.1038/s41389-022-00440-4 (PMC9596416; doi:10.1038/s41389-022-00440-4)

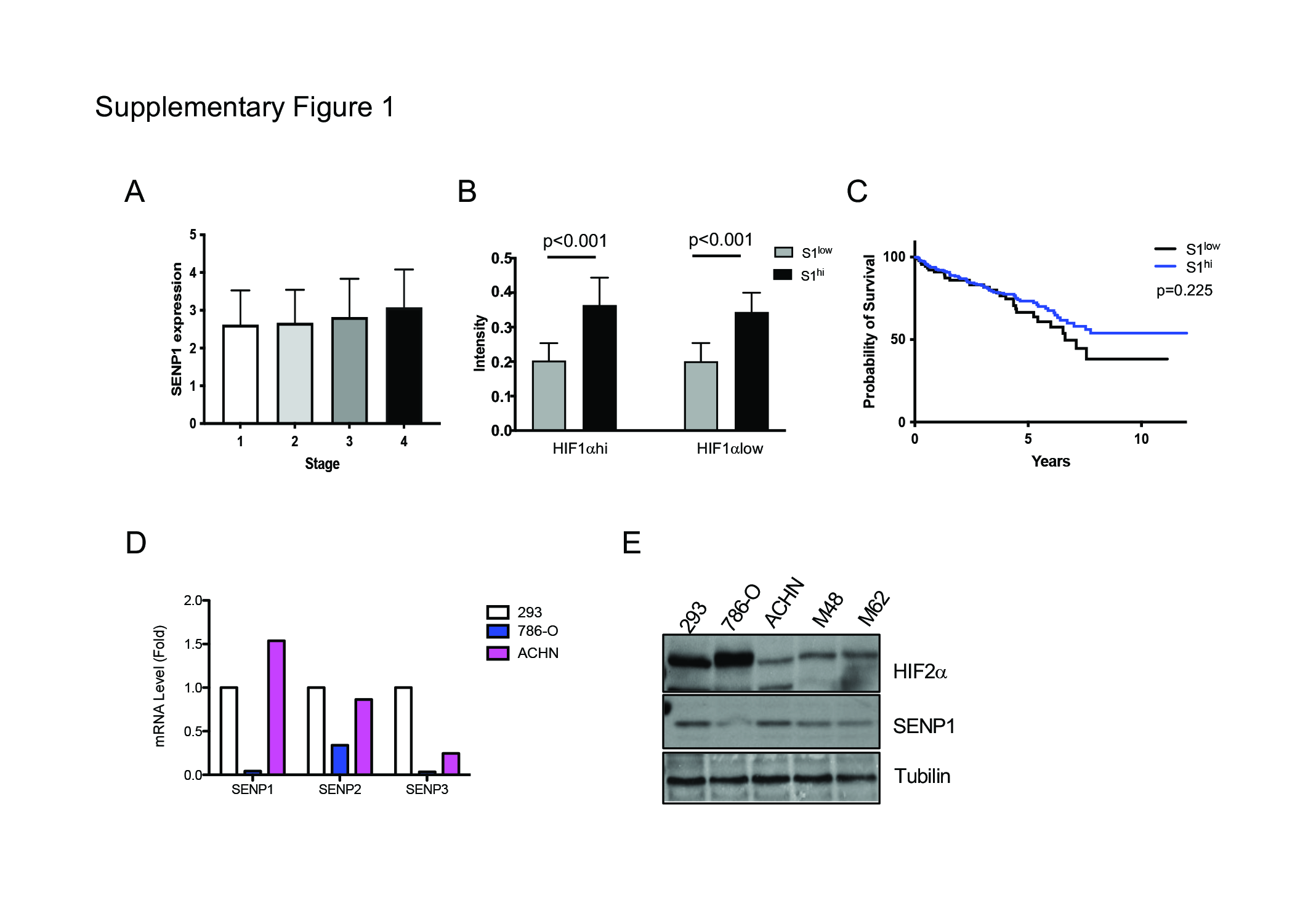

Supplement: Supplementary file 2 — Supplementary Fig 1 [file 41389_2022_440_MOESM2_ESM.tif]

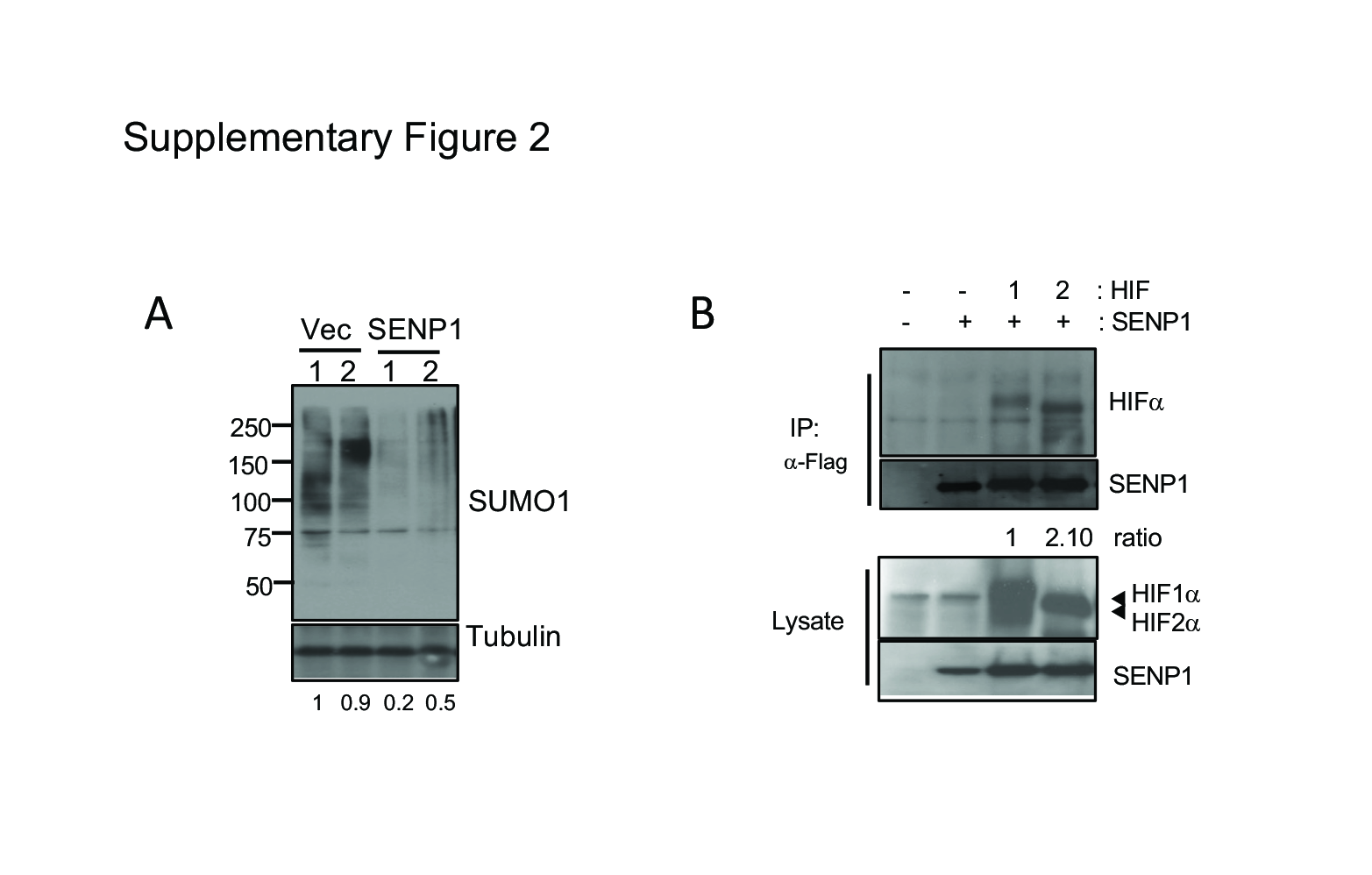

Supplement: Supplementary file 3 — Supplementary Fig 2 [file 41389_2022_440_MOESM3_ESM.tif]

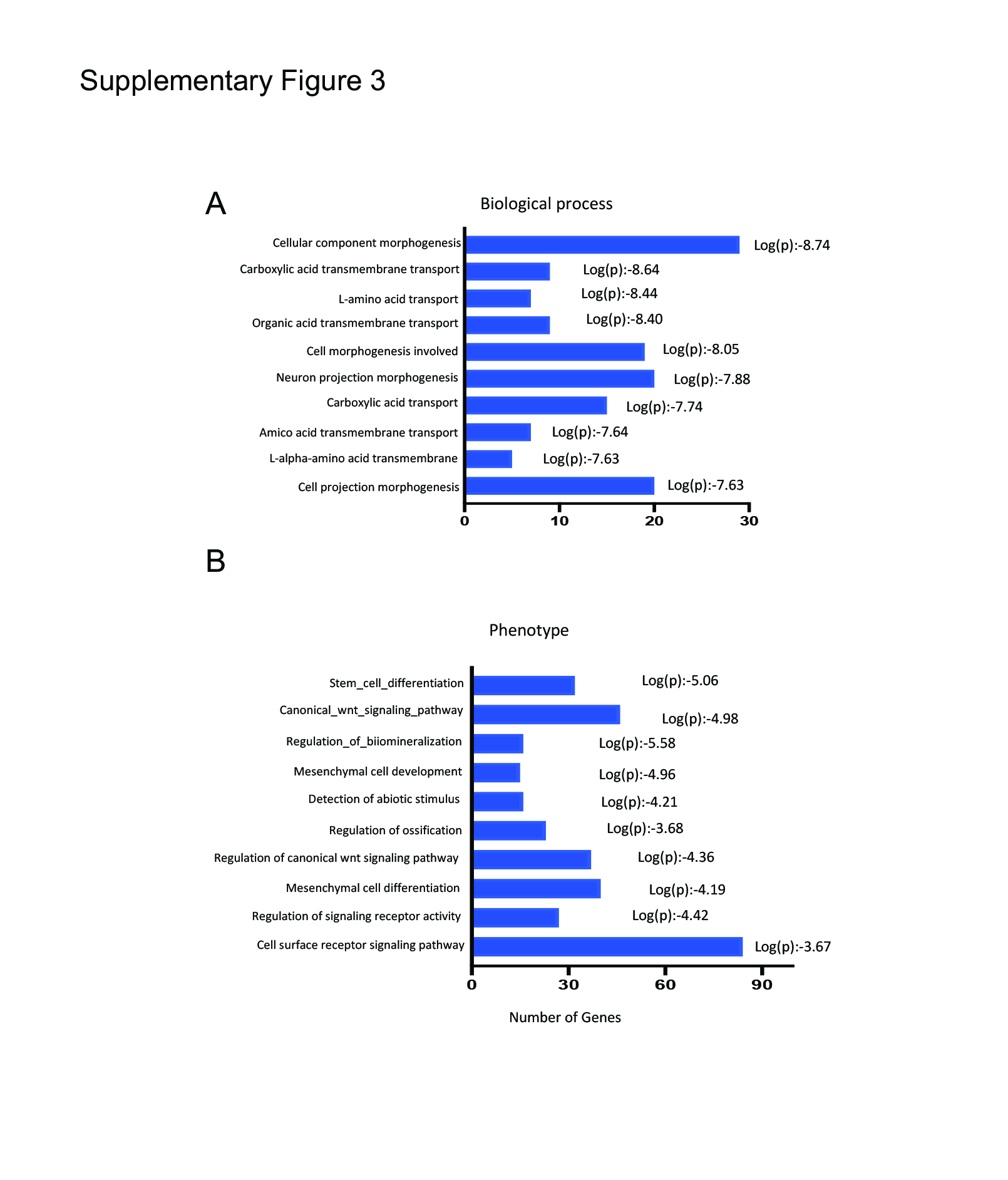

Supplement: Supplementary file 4 — Supplementary Fig 3 [file 41389_2022_440_MOESM4_ESM.tif]

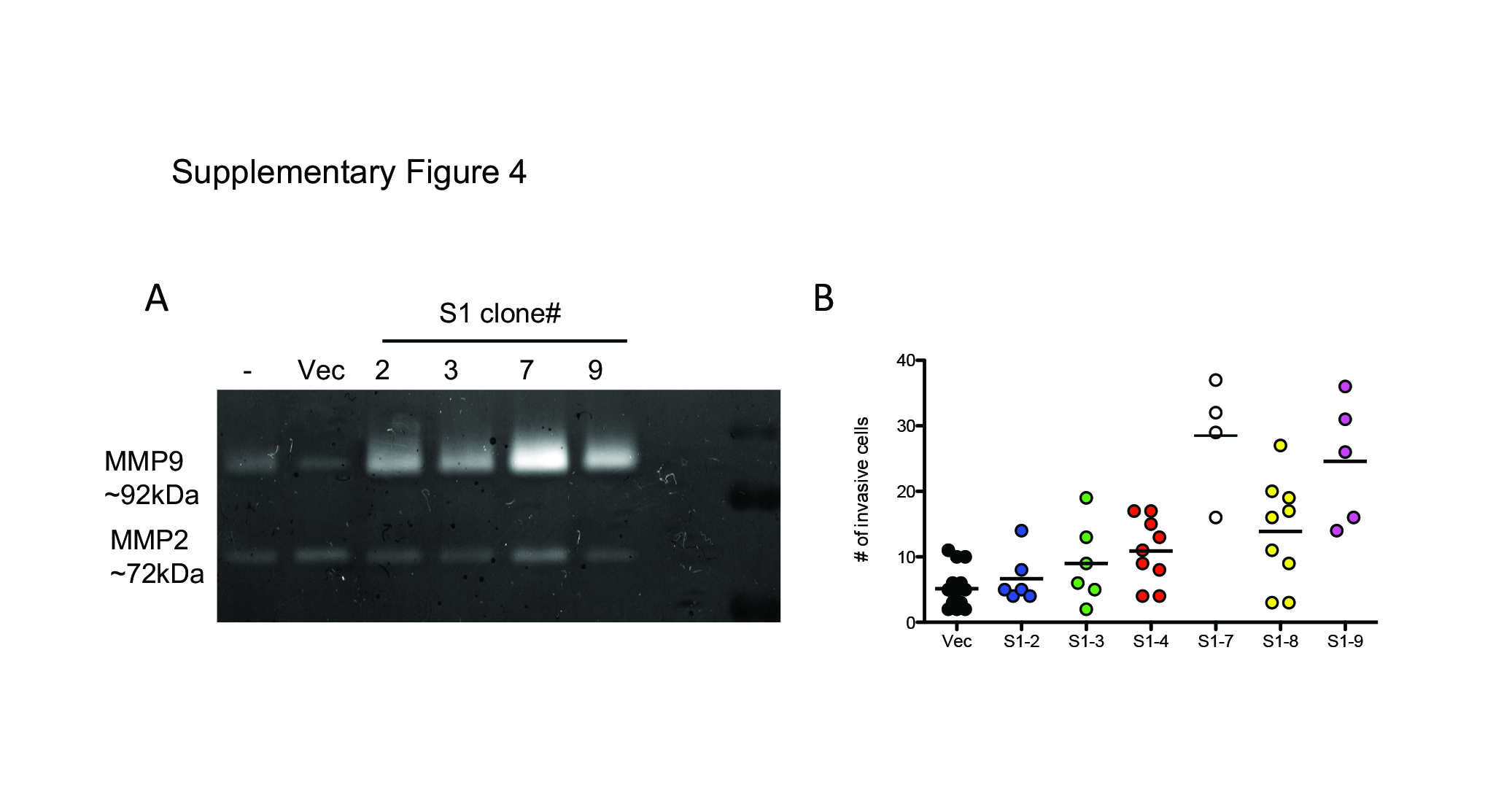

Supplement: Supplementary file 5 — Supplementary Fig 4 [file 41389_2022_440_MOESM5_ESM.tif]

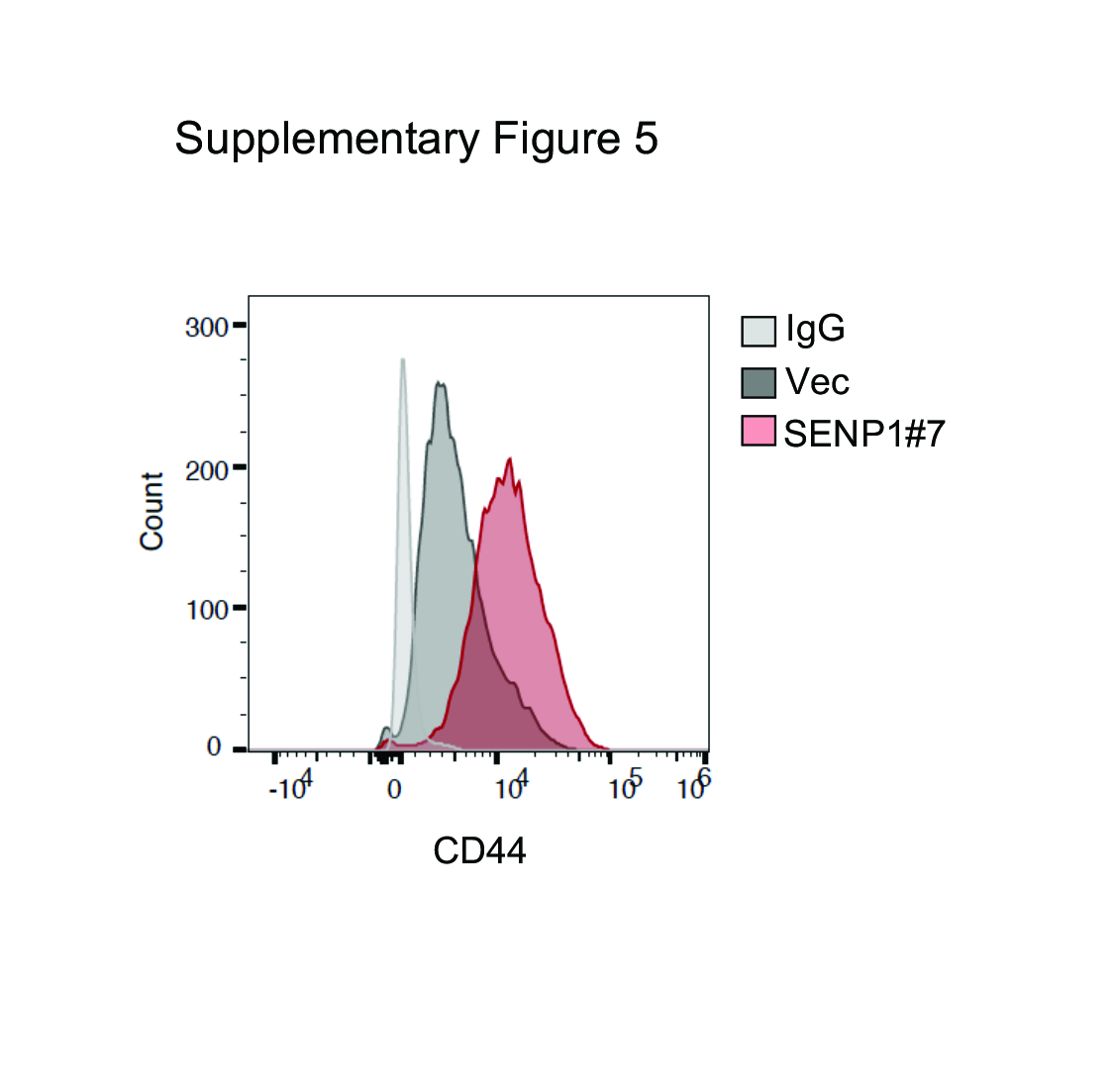

Supplement: Supplementary file 6 — Supplementary Fig 5 [file 41389_2022_440_MOESM6_ESM.tif]

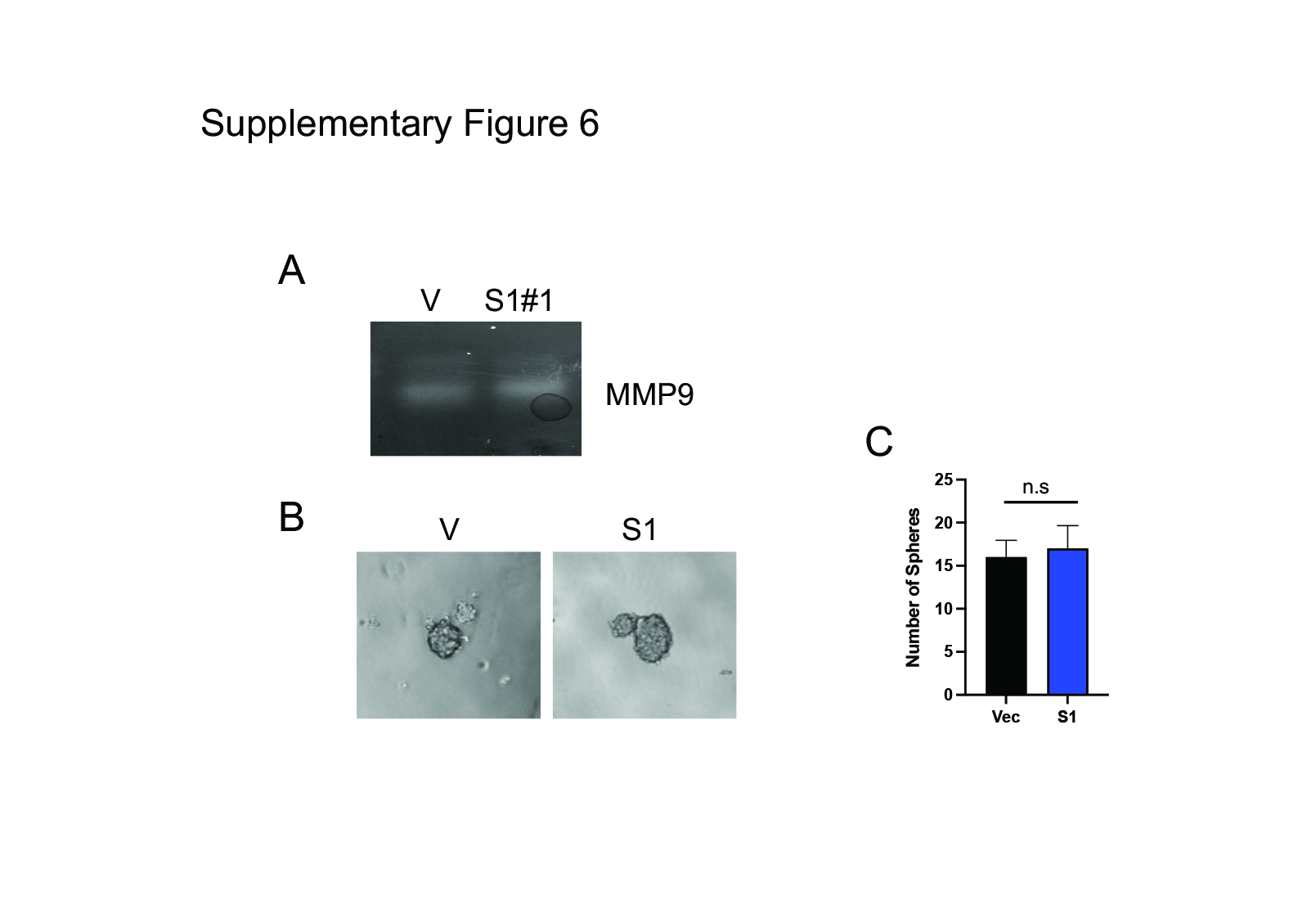

Supplement: Supplementary file 7 — Supplementary Fig 6 [file 41389_2022_440_MOESM7_ESM.tif]

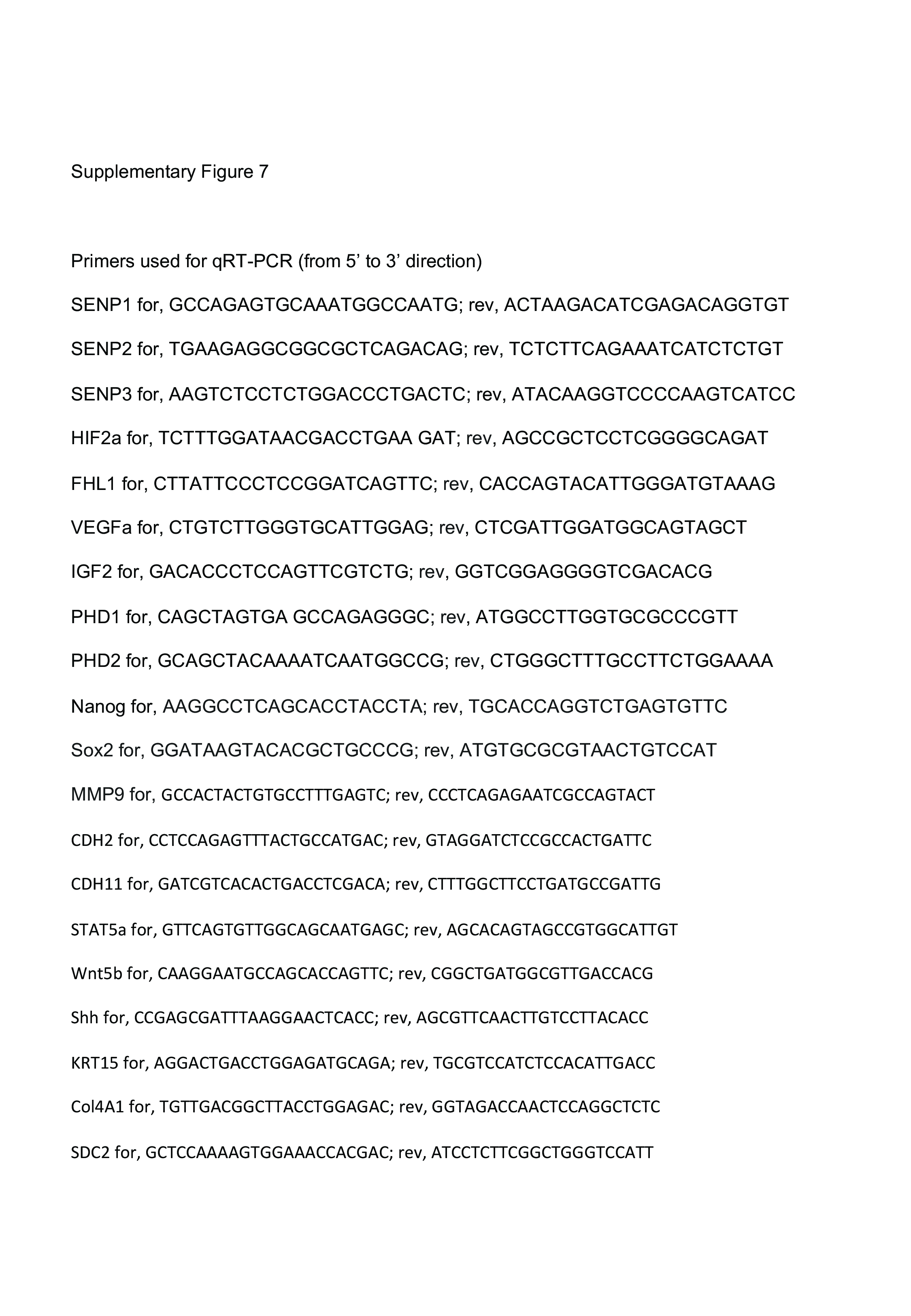

Supplement: Supplementary file 8 — Supplementary Fig 7 [file 41389_2022_440_MOESM8_ESM.tif]
